# The influence of lifestyle changes (diet, exercise and stress reduction) on prostate cancer tumour biology and patient outcomes: A systematic review

**DOI:** 10.1002/bco2.237

**Published:** 2023-04-06

**Authors:** Zach Dovey, Amir Horowitz, Nikhil Waingankar

**Affiliations:** ^1^ Mount Sinai Health System, Department of Urology Icahn Medical School New York New York USA; ^2^ Icahn School of Medicine The Mount Sinai Hospital New York New York USA

**Keywords:** biomarkers, cancer outcomes, diet, mental health, physical exercise, prostate cancer, stress reduction

## Abstract

**Background:**

The mostly indolent natural history of prostate cancer (PCa) provides an opportunity for men to explore the benefits of lifestyle interventions. Current evidence suggests appropriate changes in lifestyle including diet, physical activity (PA) and stress reduction with or without dietary supplements may improve both disease outcomes and patient's mental health.

**Objective:**

This article aims to review the current evidence on the benefits of all lifestyle programmes for PCa patients including those aimed at reducing obesity and stress, explore their affect on tumour biology and highlight any biomarkers that have clinical utility.

**Evidence acquisition:**

Evidence was obtained from PubMed and Web of Science using keywords for each section on the affects of lifestyle interventions on (a) mental health, (b) disease outcomes and (c) biomarkers in PCa patients. PRISMA guidelines were used to gather the evidence for these three sections (15, 44 and *16* publications, respectively).

**Evidence synthesis:**

For lifestyle studies focused on mental health, 10/15 demonstrated a positive influence, although for those programmes focused on PA it was 7/8. Similarly for oncological outcomes, 26/44 studies demonstrated a positive influence, although when PA was included or the primary focus, it was 11/13. Complete blood count (CBC)‐derived inflammatory biomarkers show promise, as do inflammatory cytokines; however, a deeper understanding of their molecular biology in relation to PCa oncogenesis is required (16 studies reviewed).

**Conclusions:**

Making PCa‐specific recommendations on lifestyle interventions is difficult on the current evidence. Nevertheless, notwithstanding the heterogeneity of patient populations and interventions, the evidence that dietary changes and PA may improve both mental health and oncological outcomes is compelling, especially for moderate to vigorous PA. The results for dietary supplements are inconsistent, and although some biomarkers show promise, significantly more research is required before they have clinical utility.

## 1 INTRODUCTION

National Cancer Institute figures show there are 1.7 million men with prostate cancer (PCa) in the United States. Globally, there are over 1 million new cases annually, with incidence highest in Australasia, North America and Western and Northern Europe.^1^ The mostly indolent natural history and long survival associated with the diagnosis provides a unique opportunity for men to explore diet and lifestyle interventions to alter the trajectory of their disease. Apart from age, family history and ethnicity, modifiable environmental and dietary factors may confer increased risk. Metabolic syndrome,^2^, ^3^ diabetes,^4^ obesity,^5^ high alcohol intake,^6^ smoking^7^ and dietary constituents and supplements^8^ (fried food,^9^ cadmium,^10^ high and low vitamin D,^11^ vitamin E and selenium,^12^ lycopenes,^13^ green tea, cruciferous vegetables, pomegranate^14^ and phytoestrogens^15^) have all been found to have either negative or positive effects on PCa development or progression.^1^ Although several studies have investigated individual dietary factors, a broader approach has been to assess the influence of a reduction in obesity, body mass index (BMI) and chronic stress. The molecular biology underlying this has been explored with details of how obesity and stress affect proinflammatory cytokines, growth factors and the immune response both systemically and within the emerging tumour microenvironment (TME).^16^, ^17^

Evidence from these studies would suggest appropriate changes in lifestyle including diet, exercise and stress reduction may improve both disease outcomes and patient's mental health^18^ as well as reducing the economic cancer burden on global health systems.^19^ Moreover, there may be biomarkers that have utility for clinicians and patients to assess whether these lifestyle changes are having a positive benefit.^20^

As research into lifestyle programmes gains momentum, this article aims to review the current evidence on the benefits of lifestyle programmes for PCa patients aimed at reducing obesity and stress as well as other dietary modifications and supplements, explore how tumour biology may be affected by such interventions and highlight any biomarkers that have clinical utility.

## 2 OBESITY, SYSTEMIC INFLAMMATION AND PCa TUMOUR BIOLOGY

Obesity may promote the development and progression of several cancers, including prostate, via the induction of systemic inflammation and its local influence on the TME.^21^ In a state of obesity, adipose tissue exceeds its blood supply leading to tissue hypoxia, triggering a chain of events ultimately causing sustained inflammatory signalling. Local ischaemia activates the cytokine MCP‐1, causing macrophage proliferation and coalescence resulting in crown like structures around dying adipose cells.^22^ After macrophage phagocytosis, released free fatty acids stimulate toll like receptor 4 and nuclear factor kappa B, with increased expression of proinflammatory genes cyclooxygenase 2 (COX‐2), TNF‐alpha and IL‐1Beta.^23^ Further release of free fatty acids with associated TNF‐alpha and IL‐6 cytokine signalling results in sustained adipose inflammation. Elevated circulating levels of TNF‐alpha and IL‐6 result in a switch to a systemic pro‐inflammatory state, with adipose tissue in obese patients providing a source of these pro‐inflammatory mediators for a developing TME. In PCa, IL‐6 is also known to activate the androgen receptor and so may specifically promote PCa cell proliferation and survival.^24^ Obesity‐induced insulin resistance is also associated with elevating circulating levels of insulin like growth factor (ILGF) that may promote carcinogenesis as may other signalling pathways associated with obesity such as the adipocyte specific hormone leptin (stimulating cell proliferation), adipokines (increasing TNF‐alpha secretion), reduced adiponectin (anticancer activity by inhibition of vascular endothelial growth factor [VEGF] and increased insulin sensitivity), ceruloplasmin (promoting angiogenesis) and the kynurenine pathway (activating the aryl hydrocarbon receptor [AHR] with suppressed T cell effector functioning and increased Treg activity).^25^

Clinical studies have also investigated the association between obesity and PCa.^26^ Examining tumour and benign tissue in overweight and normal weight men with PCa, nearly 95% of whom had localised disease, 15 gene sets were found to be overexpressed in obese men, 5 of which functioned for chromatin modification and remodelling, which are linked to DNA mutation burden. Moreover, obese patients had worse Gleason grades and a poorer prognosis independent of disease stage.^26^

Another study, investigating weight loss in presurgical PCa patients, found weight loss‐induced mixed effects on tumour gene expression, with increased expression of the proliferative marker Ki67 in malignant epithelium as well as reduced expression of genes related to insulin secretion (EFNA5), and increased expression of immune response genes (MRC1, HLA‐PB1 and CD86) and DNA repair genes.^27^ The genetic changes affecting insulin metabolism, immune responses and DNA repair are biologically advantageous, but elevation of Ki67 in tumour tissue is not. This may be related to the ‘Obesity paradox’ seen in advanced disease when significant tumour burden and the catabolic state results in obesity and excess adipose tissue providing a biological advantage.^28^ A follow‐up study from the same group noted weight loss was associated with loss of lean muscle and muscle catabolism, activating mitochondrial rather than glycolytic pathways. The increased Ki67 was highly linked to loss of lean muscle mass and so could potentially be reversed by combining lifestyle programmes with both weight loss and resistance muscle training to maintain lean muscle.^29^

Another clinical trial evaluated the effect of exercise in obese patients with advanced PCa on circulating tumour cells (CTCs). CTCs are of prognostic value in advanced PCa, and platelets have been shown to interact with CTCs in a process known as ‘cloaking’ that facilitates immune escape from NK cells.^30^ A positive correlation was found between CTC and platelet count, which persisted in control and overweight groups but less so in the exercise group and not at all in the normal weight group. These results may give insight into the role of obesity in disease progression by a platelet cloaking CTC mechanism,^31^ although whether this is relevant to localised and locally advanced disease requires further study.

## 3 STRESS RESPONSES AND PCa TUMOUR BIOLOGY

Chronic stress has been implicated in the development of several cancers, in part by similar mechanisms to obesity. Through activation of the sympathetic nervous system (SNS) and the hypothalamic–pituitary–adrenal (HPA) axis, chronic stress can not only promote oncogenesis but also produce systemic inflammation and suppress the immune system. The proposed oncogenic effect of catecholamines is mediated by the Beta 2 adrenergic receptor (ADRB2) enhancing tumour growth and angiogenesis by activation of the c‐AMP protein kinase A (PKA) pathway with subsequent elevation of matrix metalloproteinases and VEGF, as well as p53 breakdown and DNA damage.^32^ Additionally, Scr phosphorylation by the same pathway activates Ras‐related protein 1 (Rap1) that inhibits extracellular signal regulated kinases (ERKs) promoting tumour cell invasion and migration.^33^ By elevating corticosteroids and catecholamines, chronic stress may stimulate pro‐tumorigenic immune cells to produce IL‐10, IL‐6, TNF‐alpha and MCP‐1 as well as activating the COX‐2/PGE2 pathway to produce VEGF, all of which may influence TMEs to suppress tumour immunity. Moreover, a reduction in IL‐12 and elevation of IL‐10 selectively inhibit Th1, subsequently reducing cytotoxic T lymphocytes (CTL)‐mediated interferon production and cellular immunity.^32^ Bio‐behavioural studies of stress have also demonstrated its influence on gene expression in the TME with stress‐induced activation of the SNS inhibiting programmed cell death and the immune response as well as promoting angiogenesis, inflammation, epithelial to mesenchymal transformation and tumour invasion.^34^, ^35^, ^36^, ^37^

A cancer diagnosis itself increases stress levels and so may potentially exacerbate disease progression by the above mechanisms.^38^ Specific to PCa, an early clinical trial, albeit with small numbers, investigated whether 6 months of mindfulness‐based stress reduction (MBSR) (with a plant‐based diet) could influence prostate specific antigen (PSA) levels in localised PCa patients following surgery or radiotherapy.^39^ Of the 14 patients recruited, 8 had intermediate or high‐risk disease, and with an average time from treatment to trial recruitment of 43 months, a significant reduction in post intervention PSA levels and PSA doubling times was seen compared with patients' PSA dynamics before the intervention.

A similar study investigating MBSR in a lifestyle intervention also including diet and physical activity (PA) examined changes in prostate gene expression in a cohort of men under active surveillance (AS) for low‐risk PCa.^40^ In the 30 recruits, there was reduced expression of 453 genes and increased expression of 48 genes all relating to tumorigenesis, including intracellular protein transport, phosphorylation and metabolism as well as down regulation of RAN and SHOC2 oncogenes. However, with both studies, separating the influence of MBSR from the dietary and exercise aspects of the lifestyle intervention is difficult.

The influence of stress on tumour biology and the racial disparity seen in PCa incidence and outcomes has also been investigated.^41^ Studies have shown from early in life, children raised in a low socioeconomic environment develop resistance to glucocorticoid signalling, leading to exaggerated inflammatory and adrenocortical responses, which may then contribute to oncogenesis as adults.^42^ Later in life, chronic stress induced by racist behaviour may give rise to increased b‐adrenergic and corticosteroid signalling,^43^ increased expression of pro‐inflammatory genes^44^ and increased smoking and alcohol consumption^45^ creating an environment that also promotes cancer incidence and progression.^41^

## 4 EVIDENCE ACQUISITION/METHODS

**FIGURE 1:**
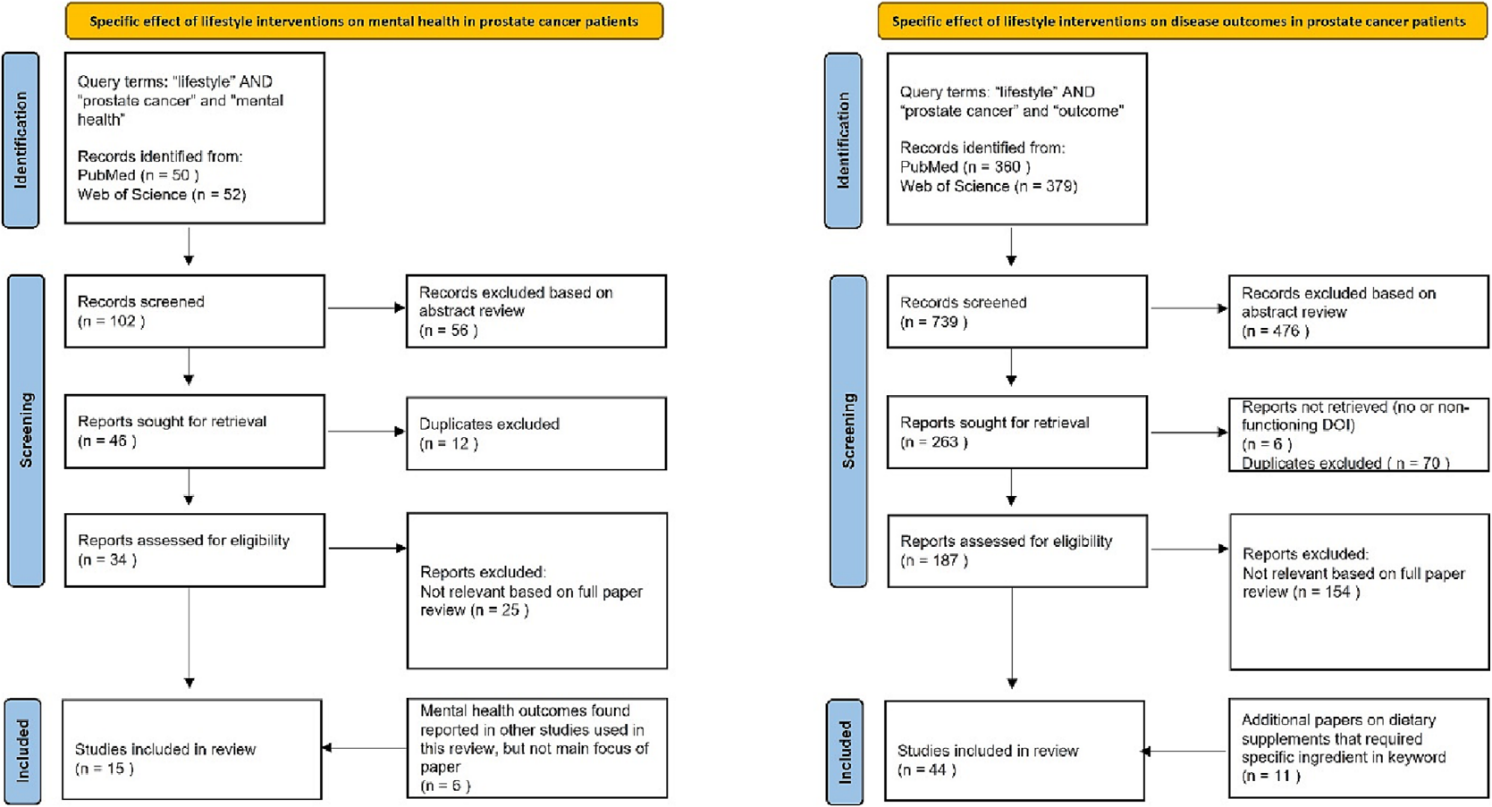
PRISMA flow diagram for the influence of Lifestyle Interventions Mental Health and Oncological Outcomes Literature Search.

**FIGURE 2:**
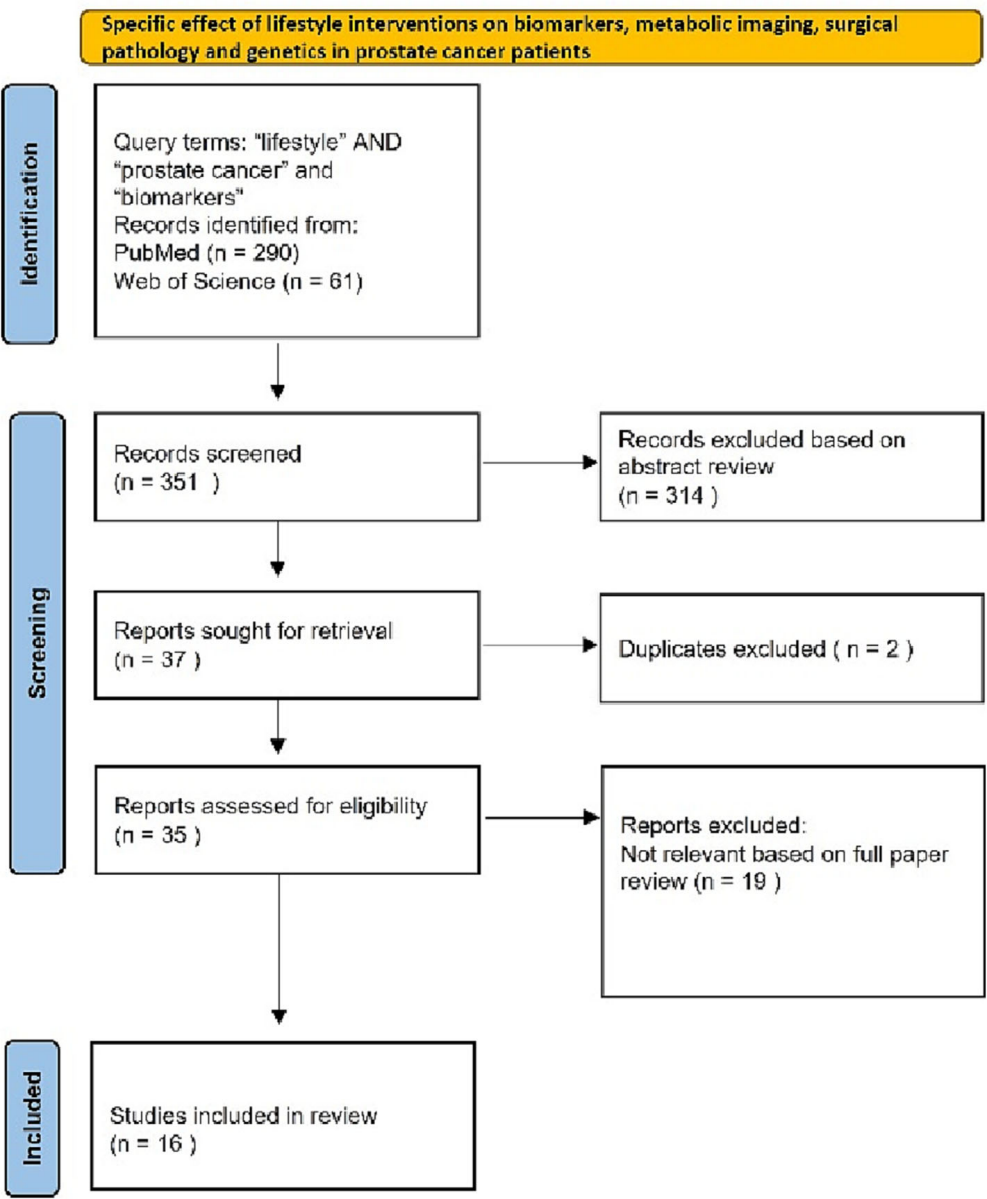
PRISMA flow diagram for the influence of lifestyle interventions on the Complications of Treatment and Biomarkers and Metabolic Imaging Literature Search.

Evidence for this review was obtained from PubMed and Web of Science using keywords specific to each section on the effects of lifestyle interventions on (a) mental health, (b) disease outcomes and (c) biomarkers, metabolic imaging, surgical pathology and genetics in PCa patients. The search strategy aimed to include all potential terms that may be used to describe a ‘Lifestyle Program’ focused on any aspect of diet (including supplements), PA and MBSR, as well as their influence on mental health outcomes, oncological outcomes, biomarkers and tumour biology. PRISMA guidelines were used to gather the evidence for these three sections. All publications including systematic reviews, qualitative, quantitative, experimental and mixed method design studies were included. References of the chosen articles were also screened and relevant missing studies added. Any article not available in English was excluded. The PRISMA flow charts are shown in Figures 1 and 2, and the PRISMA checklist is provided in the Supporting Information. Abstracts and full papers were reviewed by two qualified individuals by title, abstract and then complete manuscript to avoid selection bias. The data were then extracted from the chosen articles as per Tables 1, 2, 3 and analysed in the text accordingly.

**TABLE 1:** Summary of different studies on effect of lifestyle interventions in PCa patients on mental health outcomes. A total of 15 different studies with ~3000 participants.

Reference	Cohort size and demographics	Localised/advanced PCa	Type and duration of lifestyle intervention	Mental health outcomes	Was the intervention successful in improving mental health?
Carli et al.^46^	425 participants; 213 in NEVERMIND intervention, 212 in control Mean age = 59.41 years ~24% prostate cancer patients (100 patients)	Stages II, 11 and IV % not available	Lifestyle—a smart shirt and mobile app solution for depression self‐management. Duration: 12 weeks.	Primary outcome was depressive symptoms @ 12 weeks measured by Beck Depression Inventory II Intervention group had statistically significantly lower depressive symptoms @ 12 weeks (*p* < 0.001)	Lifestyle intervention successful (personalised based on feedback from smart wearable technology and app)
Conroy et al.^47^	349 female (83% breast cancer survivors) 292 male (89% prostate cancer survivors) Mean age all participants = 73.1 years	% not available	Physical activity (PA) RENEW trial secondary analysis focused on obese patients Duration: 12 months	Quality of Life (QoL) from Short Form 36 Health Status Survey (SF‐36), specifically the sub‐scales Emotional Role Functioning, Vitality, Mental Health and Social Role Functioning. For men, moderate to vigorous physical activity was positively linked with Mental Health, Emotional Role Functioning and Vitality	PA intervention successful for moderate to vigorous activity (>3 MET hours per week)
Farris et al.^48^	817 prostate cancer survivors Mean age at PCa diagnosis = 67.3 years	Localised 74.1% Advanced 15.9%	Physical activity (PA) Duration: levels of physical activity were assessed a 3 time points over a period of 36 months from diagnosis	Quality of Life (QoL) from Short Form 36 Health Status Survey (SF‐36), both Physical and Mental Component Summaries Patients who met PA guidelines consistently had significantly higher physical QoL scores compared with those who did not. Those that met guidelines had improved QoL, whereas those who did not maintain intervention indicated a decreased QoL.	PA intervention successful for moderate to vigorous activity (150 min moderate or 75 min vigorous per week)
Mosher et al.^49^	753 cancer survivors; 42% prostate cancer (319 patients) Mean age all survivors = 73 Mean time since diagnosis = 9 years	For the whole cohort‐ Localised 69% Advanced 27% Unknown 4%	Lifestyle—physical activity (PA), BMI, Healthy Eating Index Duration: cross sectional analysis assessing above baseline lifestyle parameters for the RENEW trial focused on older survivors (>65 years) >5 years from diagnosis.	Quality of Life (QoL) from Short Form 36 Health Status Survey (SF‐36), both Physical and Mental Component Summaries Greater weekly minutes of exercise were associated with better physical QoL, physical functioning, vitality and better social functioning.	Lifestyle intervention largely unsuccessful for mental health (social functioning the exception)
Taylor et al.^50^, ^51^	134 prostate cancer patients on androgen ablation therapy Mean age = 69.2 Mean therapy time = 32.7 months	Advanced 100%	Physical activity (PA) group and an educational support (ES) and standard care group Duration: 6 months	Quality of Life (QoL) from Short Form 36 Health Status Survey (SF‐36), both Physical and Mental Component Summaries, Centers for Epidemiologic Studies‐Depression (CES‐D) and State/Trait Anxiety Inventory scale (STAI) No significant differences were found between groups on primary QOL outcomes following the 6‐month interventions. Both group programmes did benefit patients with lower psychosocial functioning at baseline. Patients with lower mental health and social support scores had significant improvements in these measures compared with standard care.	PA intervention largely unsuccessful (some evidence of success for those patients with limited psychosocial functioning)
Daubenmier et al.^52^	93 prostate cancer patients managed by active surveillance Mean age = 64.8 years (intervention group); 66.5 years (control group)	Localised 100%	Lifestyle—diet (low fat vegan), physical activity (PA) (3 h per week of moderate exercise) and stress management (1 h per week). Duration: 12 months	Quality of Life (QoL) from Short Form 36 Health Status Survey (SF‐36), both Physical and Mental Component Summaries, the Perceived Stress Scale and the Sexual Function subscale of the UCLA Prostate Cancer Index. Compared with controls after 12‐month follow up, intervention cohort had significantly improved their lifestyle. In both groups, those who reported a healthier lifestyle also reported better physical and mental HR‐QOL and sexual function. For participants whose lifestyle improved, an improved physical HR‐QOL and lower perceived stress was observed.	Lifestyle intervention successful
Baguley et al.^53^	23 men Mean age 65.9 years Receiving ADT for ≥3 months	Advanced 100%	Lifestyle—Mediterranean diet and high intensity interval (HIIT) training Duration: 20 weeks	Cancer‐related fatigue and quality of life were measured using the Functional Assessment of Chronic Illness Therapy: Fatigue (FACIT‐F), FACIT‐general (FACIT‐G) questionnaire and the Medical Outcomes Study 36‐Item Short‐Form Health Survey (SF‐36). The lifestyle intervention increased cardiorespiratory fitness and reduced body weight. Improvements were seen in quality of life and cancer‐related fatigue after 20 weeks.	Lifestyle intervention successful
Pollock et al.^54^	48 men who had started ADT <6 months Median age 66 years	Advanced 100%	Lifestyle—counselling on exercise, nutrition and symptom management Duration: 12 months	Outcomes were measured by Patient Health Questionnaire (PHQ‐9; depression screening), Attention Functional Index (AFI; cognitive function), Lee Fatigue Scale, International Prostate Symptom Score (IPSS; lower urinary tract symptoms), Expanded Prostate Cancer Index Composite Short form (EPIC‐26; erectile, urinary, bowel and hormonal function), Hot Flash Related Daily Interference Scale 27 and 12‐Item Short Form Survey (SF‐12‐V2; overall quality of life). No differences observed between intervention and usual care cohort in any of the measured outcomes.	Lifestyle intervention unsuccessful
Ashton et al.^55^	40 PCa patients following RARP Mean age = 64.6 years	Localised 100%	Physical activity—resistance exercise training in 3 weekly sessions using resistance bands Duration: 6 months	Questionnaires were used to assess QoL (EQ‐5D‐5L and FACT‐P), fatigue (Brief Fatigue Inventory) and self‐reported exercise behaviour (modified Godin Leisure Time Exercise Questionnaire). RET is an effective type of training that provides beneficial effects on HR‐QoL (as well as on aerobic exercise capacity and muscular strength).	PA intervention successful
Dieperink et al.^56^	161 PCa patients, treated with (majority 96%) ADT or radiotherapy Mean age = 68.2 years	Localised 100%	Lifestyle—Psychosocial support from nurses and counselling in pelvic floor exercises Duration: 20 weeks	QoL arising from the Medical Outcome Study Short‐form‐12 (SF‐12). While the intervention was successful in improving the physical composite score of the SF‐12, no difference was observed for the mental composite score	Lifestyle intervention unsuccessful
Dieperink et al.^57^	3‐year follow up to above study 143 respondents	Localised 100%	Lifestyle—psychosocial support from nurses and counselling in pelvic floor exercises Duration: 20 weeks	QoL arising from the Medical Outcome Study Short‐form‐12 (SF‐12). No difference in mental outcomes after 3‐year follow up.	Lifestyle intervention remains unsuccessful
Mardani et al.^58^	71 PCa patients Mean age = 69.4 years	Localised 100%	Physical activity 12‐week exercise programme—1 session of group exercise and 3 sessions of individual exercise per week using community facilities Duration: 12 weeks	QLQ‐C30 survey is used to assess QoL in patients with cancer. It measures five functional domains and nine domains of symptoms, global health status and QoL. The functional domain includes physical, role, emotional, cognitive and social aspects. Symptoms include fatigue, nausea and vomiting, pain, dyspnoea, insomnia, appetite loss, constipation and diarrhoea. In addition, it asks about financial difficulties, and a separate domain is for the global health status and QoL. Improvements in physical, role, emotional, social and sexual functions were reported.	Physical activity intervention was largely successful.
Kang et al.^59^	52 PCa patients undergoing active surveillance	Localised 100%	Physical activity—3 times per week supervised HIIT programme Duration: 12 weeks	Patient‐reported outcomes consisted of fear of cancer progression (Fear of Cancer Recurrence Inventory), PCa‐specific anxiety (Memorial Anxiety Scale for Prostate Cancer), quality of life (European Organization for Research and Treatment of Cancer Quality of Life Questionnaire Core), PCa symptoms (Expanded Prostate Cancer Index Composite) and psychological health outcomes (e.g., fatigue, stress and self‐esteem) Intervention significantly improved total prostate cancer‐specific anxiety (*p* = 0.024), fear of progression subscale (*p* = 0.013), perceived stress (*p* = 0.037), fatigue (*p* = 0.029), hormonal symptoms (*p* = 0.005) and self‐esteem (*p* = 0.007).	Physical activity intervention was successful
Bjerre et al.^60^	214 PCa patients Mean age = 68.4	Localised 78% Advanced 21% Unknown 1%	Physical activity Training twice a week at a local football club at no cost. Duration: 6 months.	QoL was measured with the FACT‐P questionnaire and mental health determined by the Mental Component Summary of Short Form‐12. Intervention group showed improvement on the Mental Component Summary (*p* = 0.048) and mental health scale (*p* = 0.020) than standard of care participants	Physical activity intervention was successful
Hojan et al.^61^	54 PCa patients prior and after undergoing radiotherapy Mean age = 68.5	Localised 100%	Physical activity—moderate level physical activity 5 days per week Duration: 8 weeks	The QLQ‐C30 was the main instrument used to measure patient QoL and mental health outcomes (see above under the Mardani et al. entry) After RT, there was a significant improvement in emotional and cognitive functioning (*p* < 0.05) in the intervention group	Physical activity intervention was successful

Abbreviations: ADT, androgen deprivation therapy; BMI, body mass index; MET, metabolic equivalent; PCa, prostate cancer.

**TABLE 2:** Summary of 44 research studies looking at the influence of various dietary, physical activity and lifestyle interventions on prostate cancer oncological outcomes comprising over 36 000 total participants.

Reference	Cohort size and demographics	% localised/advanced PCa	Type and duration of lifestyle intervention	Health outcomes	Was the intervention successful (and at what)?
Frattaroli et al.^62^	93 men with early stage prostate cancer undergoing active surveillance	Localised 100%	Intervention—diet (low fat vegan), physical activity (PA) (3 h per week of moderate exercise) and stress management (1 h per week). Duration: 12 months	27% of control patients yet but 5% of patients subscribing to the intervention had undergone conventional prostate cancer treatment (radical prostatectomy, androgen deprivation or radiotherapy, *p* < 0.05)	Yes (PCa disease progression)
Saxe et al.^63^	10 men diagnosed with prostate cancer	Localised 100% (Post surgery with BCR)	Lifestyle intervention—plant‐based diet with stress management training) Duration: 6 months	The rate of PSA increase decreased in 8 of 10 men, while 3 had a decrease in absolute PSA. A significant decrease in the rate of increase in the intervention period (*p* = 0.01) was observed.	Yes (PSA levels and doubling times)
Richman et al.^64^	1455 men	Localised 87% Advanced 13%	Physical activity (PA) Sub analysis of the CaPSURE Study recruits who completed physical activity and dietary questionnaires 2004–2005 Duration: cross sectional design with questionnaires on average 27 months after diagnosis and average Urology follow up 22 months after that.	Men who walked briskly for ≥3 h/week had a 57% lower rate of progression compared with men who walked at an easy pace for <3 h/week (*p* value = 0.03). Walking pace was associated with decreased risk of progression independent of duration (*p* value = 0.01). Brisk walking after diagnosis may delay or inhibit prostate cancer progression.	Yes (prostate cancer progression defined by prostate cancer mortality, bone metastases from prostate cancer, biochemical recurrence or secondary treatment)
Bonn et al.^65^	4623 men diagnosed with localised prostate cancer	Localised 100%	Physical activity (PA) Duration: cross sectional design with study performed on men diagnosed with PCa between 1997 and 2002 and followed up to 2012.	For PCa‐specific mortality, statistically significantly lower rates were observed for men walking/bicycling ≥20 min/day or exercising ≥1 h/week	Yes (PCa and overall mortality)
Kenfield et al.^66^	2705 men diagnosed with localised prostate cancer	Localised 100%	Physical activity (PA) Duration: sub analysis of Health Professions Follow up study on mean diagnosed PCa between 1990 and 2008 with PA assessed formally every 2 years. F/U 27 years.	Men who were physically active had lower risk of all‐cause mortality (*p* = <0.001) and PCa mortality (*p* = 0.04). Both non‐vigorous activity and vigorous activity were associated with significantly lower overall mortality. Men exercising vigorously before and after diagnosis had the lowest risk.	Yes (Overall and PCa mortality)
Kucuk et al.^67^	26 men diagnosed with localised prostate cancer	Localised 100%	Dietary supplementation—15 mg of lycopene 3 weeks prior to prostatectomy (*n* = 15) or no supplementation (*n* = 11) Duration: 3 weeks	Men in the intervention group had smaller tumours, reduced involvement of surgical margins and/or extra‐prostatic tissues with cancer (73% vs. 18%, organ‐confined disease) and less diffuse involvement of the prostate by high‐grade prostatic intraepithelial neoplasia (33% vs. 0%, focal involvement) in comparison with those in the control group. Mean plasma PSA levels were lower in the intervention group compared with the control group.	Yes (tumour size, surgical margins, PSA levels)
Dalais et al.^68^	29 men prostate cancer patients scheduled to undergo a radical prostatectomy	Localised 100%	Dietary—high phytoestrogen (soy, soy & linseed) diets Duration: 24 days	Significant differences were detected between the control wheat group and the intervention group for percentage change in total PSA (−12.7%; *p* = 0.02) and the percentage change in free/total PSA ratio (27.4% vs. −15.6%, *p* = 0.01). Evidence suggests those who consume high phytoestrogen diets have a reduced risk of PCa development and progression.	Yes (change in PSA and free/total PSA ratio)
Hussain et al.^69^	41 PCa patients with rising PSA levels	Localised 66% Advanced 34%	Dietary—soy isoflavones supplementation Duration: 5.5 months	Stabilisation of the PSA occurred in 83% of patients in the group that had increasing PSA following localised therapy and 35% of those currently receiving hormone therapy. There was a decrease in the rate of the rise of serum PSA in the whole group (*p* = 0.01) with rates of rise decreasing from 14% to 6% in group following localised therapy (*p* = 0.21) and from 31% to 9% in group III currently undergoing hormone therapy following the soy isoflavone intervention.	Yes (rate of rise of PSA levels)
Jarred et al.^70^	38 patients upon diagnosis of prostate cancer pre radical prostatecomy	Localised 100%	Dietary—isoflavones from red clover Duration: 20 days	Apoptosis in radical prostatectomy specimens from treated patients was significantly higher than in control patients (*p* = 0.0018). Evidence suggests that dietary isoflavones may halt the progression of prostate cancer through induction of apoptosis in low to moderate‐grade tumours.	No (apoptosis of cancer cells aside, no significant differences between Gleason score, pre‐ and post treatment serum PSA, serum testosterone, or biochemical factors in the treated patients ( *p* > 0.05 ).
Schroeder et al.^71^	49 PCa patients with rising PSA levels after radical prostatectomy (*n* = 34) or radiotherapy (*n* = 15)	Localised 100% (with BCR)	Dietary supplement—soy, isoflavones, lycopene, silymarin and antioxidants Duration: 10 weeks	An increase in the PSA doubling time from 445 to 1150 days for the supplement and placebo periods was observed. Dietary supplement used in this study was shown to delay PSA progression.	Yes (PSA doubling time)
Bourke et al.^72^	50 advanced prostate cancer patients receiving AST for a minimum of 6 months (*n* = 25) or standard care (*n* = 25). Follow up at 3 and 6 months	Advanced 100%	Lifestyle—12‐week programme comprising aerobic and resistance exercise, plus dietary advice Duration: 12 weeks	No effects on clinical prostate cancer disease biomarkers were observed.	No (change in PSA)
Richman et al.^73^	4577 men with non‐metastatic prostate cancer	Localised 100%	Dietary—animal fat versus vegetable fat intake Duration: sub analysis of Health Professions Follow up study on mean diagnosed PCa between 1990 and 2008 with dietary habits assessed formally every 2 years. F/U 27 years	Replacing animal fat and carbohydrates with vegetable fat may reduce the risk of all‐cause mortality in non‐metastatic prostate cancer patients.	Yes (PCa and all‐cause mortality)
Friedenreich et al.^74^	830 stage II–IV PCa patients with up to 17 year follow up.	Localised 93% Advanced 7%	Physical activity (PA) Duration: PCa patients recruited between 1997 and 2000 with F/U to 2014. Post recruitment PA was documented up to three times during F/U	Those who were more physically active post diagnosis or engaged in more recreational physical activity before and after diagnosis had higher survival. Recreational physical activity post diagnosis was associated with a reduced risk of PCa death.	Yes (PCa and all‐cause mortality)
Zaorsky et al.^75^	2207 patients treated with IMRT with a median dose of 78 Gy. Median follow up of 46 mo. Of these patients, 43% were low risk, 37% were intermediate risk and 20% were high risk.	Localised 100%	Dietary—health supplements or MHSs (products marketed as men's and prostate health supplements, e.g., vitamins, minerals and herbs) Duration: retrospective analysis of a cohort of PCa patients recruited 2001–2012 with median F/U 46 months	The use of MHSs is not associated with outcomes or toxicities of prostate cancer patients receiving IMRT	No (freedom from distant metastasis, freedom from biochemical failure, overall survival and cancer‐specific survival)
Farris et al.^76^	829 prostate cancer patients followed up to 19 years	Localised 100% (at diagnosis but may have developed advanced disease during follow up)	Dietary—alcohol consumption Duration: PCa patients recruited between 1997 and 2000 with retrospective analysis of questionnaires about alcohol consumption at diagnosis and 2–3 years later. F/U maximum 19 years.	Post‐diagnosis alcohol consumption was linked to increased PCa‐related mortality after PCa diagnosis.	No (PCa mortality for low or heavy drinkers pre‐diagnosis to low or non‐drinkers post diagnosis)
Brassetti et al.^77^	85 prostate cancer patients on active surveillance. Median follow up 37 months	Localised 100%	Physical activity (PA) Duration: retrospective analysis of PCa patients on AS 2006–2019, with questionnaire self‐assessing daily PA. Median F/U 37 months	Increasing levels of physical activity are associated with a significantly reduced risk of tumour progression for patients undergoing active surveillance.	Yes (risk of tumour progression)
Plym et al.^78^	12 411 genotyped men, 3005 overall prostate cancer and 435 lethal PCa cases were observed.	% not available	Lifestyle—defined by healthy weight, vigorous physical activity, non‐smoker and healthy diet. Duration: sub analysis of Health Professions Follow up study on mean diagnosed PCa between 1990 and 2008 with dietary habits assessed formally every 2 years. F/U 27 years.	For men at the highest genetic risk of PCA, adherence to a healthy lifestyle reduced the risk of metastatic disease and PCa death.	Yes (PCA mortality)
Langlais et al.^79^	Time to prostate cancer progression patients (*n* = 2056) and prostate cancer‐specific mortality patients (*n* = 2447	Localised 100%	Lifestyle—effect of post‐diagnosis inflammatory, hyperinsulinemic and insulin‐resistant diets Sub analysis of the CaPSURE Study recruits who completed physical activity and dietary questionnaires 2004–2005 Duration: cross sectional design with questionnaires on average 27 months after diagnosis and average urology follow up 22 months after that.	Insulinemic and inflammatory dietary and lifestyle behaviours are associated with risk of PCa progression.	Yes (PCa progression)
Thomas et al.^80^	110 men whose PSA level had risen in three consecutive values, >20% over the preceding 6 months. Patients already undergoing a lifestyle intervention	Localised 100% (rising PSA in men selected for AS)	Dietary—supplementation with sodium salicylate (SS) alone or SS combined with, vitamin C, copper and manganese gluconates (CV247) Duration: 12 months	No difference in outcome between the SS or CV247 (*p* = 0.92). The intervention stopped or slowed the rate of PSA progression in 40 patients (36.4%) for over 1 year.	No (PCa progression)
Burton et al.^81^	404 men, aged 50–69 years, with localised prostate cancer undergoing active surveillance	Localised 100%	Lifestyle—diet, physical activity, smoking, Duration: sub analysis of PROTECT trial utilising baselines lifestyle data. Mean duration of F/U 4.77 years.	Smoking and exercise are modifiable lifestyle factors that may be associated with PSA levels in men with localised prostate cancer under active surveillance.	Yes (PSA levels)
Eriksen et al.^82^	26 men (53–72 years) recently diagnosed with non‐aggressive prostate cancer and on active surveillance.	Localised 100%	Lifestyle—170 g/day of whole‐grain rye and 3 × 45 min/week of vigorous physical activity for 6 months with 12‐month follow up. Duration: 6 months	No effects were found on prostate cancer progression.	No (PCa progression)
Kang et al.^83^	52 men on active surveillance with localised very low risk to favourable intermediate risk PCa.	Localised 100%	3 times per week supervised high intensity interval training (HIIT) on treadmill Duration: 12 weeks	HIIT intervention increased decreased PSA levels and velocity and prostate cancer cell growth.	Yes (PSA levels, velocity)
Kucuk et al.^84^	26 men with newly diagnosed localised disease	Localised 100%	Dietary supplementation—15 mg of lycopene 3 weeks prior to prostatectomy (*n* = 15) or no supplementation (*n* = 11) Duration: 3 weeks	Significantly less involvement of surgical margins and diffuse involvement of the prostate by high‐grade prostatic intraepithelial neoplasia in intervention group.	Yes (surgical margins)
Bowen et al.^85^ Chen et al.^86^	32 patients with localised prostate adenocarcinoma	Localised 100%	Dietary supplementation—consumption of tomato sauce‐based pasta dishes for 3 weeks (30 mg of lycopene/day) before radical prostatectomy. Duration: 3 weeks	Significant decreases in mean serum PSA concentrations by 17.5% and leukocyte 8OHdG by 21.3% after intervention was observed. Resected tissues from intervention group had 28.3% lower prostate 8OHdG compared with the control group (*p* < 0.03). Cancer cell 8OHdG staining of Gleason score‐matched resected prostate sections was reduced 36.4% in mean area (*p* < 0.018) and by 40.5% in mean nuclear density (*p* < 0.005) compared with the pre‐supplementation biopsy	Yes (PSA, cancer cell proliferation)
Clark et al.^87^	36 men with biochemically relapsed prostate cancer	Localised 100% (but biochemically recurrent)	Dietary supplementation—6 consecutive cohorts of 6 patients each received supplementation with 15, 30, 45, 60, 90 and 120 mg/day lycopene for 1 year. Plasma levels of lycopene and PSA were measured at baseline and every 3 months. Duration: 12 months	Lycopene supplementation at the doses used in this study did not result in any significant response in serum PSA	No (PSA)
van Breeman et al.^88^	105 Afro‐American men, 58 diagnosed with BPH, 47 with PCa	% not available for PCa group but likely all localised	Dietary supplementation −30 mg/day of lycopene as a tomato oleoresin or placebo for 21 days prior to prostate biopsy Duration: 3 weeks	No significant changes in 8‐oxo‐deoxyguanosine (DNA oxidation product) or malondialdehyde (lipid peroxidation product) were observed in prostate tissue and plasma, respectively, as a result of lycopene intervention.	No (DNA oxidation and lipid peroxidation products)
Schwenke et al.^89^	12 patients with progressive hormone refractory PCa	Advanced 100%	Dietary supplementation—lycopene supplementation (15 mg) was given daily for 6 months. Duration: 6 months	No clinically relevant benefits were shown for patients with advanced stages of the disease as a result of the intervention.	No (PSA)
Jatoi et al.^90^	46 patients with androgen independent PCa. All were asymptomatic and had serum PSA elevation despite hormonal manipulation	Advanced 100%	Dietary supplementation—patients given a lycopene‐rich tomato supplement at a lycopene dosage of 15 mg two times per day Duration: 4 months	Lycopene did not appear effective for androgen‐independent prostate cancer, at least as prescribed in this study.	No (PSA)
Ansari and Gupta^91^	20 patients previously treated with hormonal therapy now with clinical and biochemical evidence of disease progression	Advanced 100%	Dietary supplementation—10 mg/day of lycopene was administered for a period of 3 months Duration: 3 months	With PSA response as the main endpoint, intervention appeared effective and safe in the treatment of HRPC. It slowed or eliminated rising PSA and improved, bone pain and lower urinary tract symptoms.	Yes (PSA levels)
Henning et al.^92^	113 men diagnosed with prostate cancer scheduled for radical prostatectormy	Localised 100%	Dietary supplementation—6 cups daily green tea, black tea or water. Duration: 1 month	Evidence of a systemic antioxidant effect was observed (reduced urinary 8OHdG) only with green tea consumption. Green tea also resulted in a small yet significant decrease in serum prostate‐specific antigen (PSA) levels.	Yes (PSA levels and antioxidant effects)
McLarty et al.^93^	26 men scheduled for radical prostatectomy	Localised 100%	Dietary supplementation—daily doses of Polyphenon E, equivalent to of 1.3 g of tea polyphenols until time of radical prostatectomy. Duration: median ~ 1 month	Results showed a significant reduction in serum levels of PSA, VEGF and HGF in men with PCa after treatment with EGCG (Polyphenon E), with no elevation of liver enzymes.	Yes (PSA, HGF and VEGF levels)
Choan et al.^94^	19 patients with hormone refractory PCa	Advanced 100%	Dietary supplementation—green tea extract capsules at a dose level of 250 mg twice daily Duration: 2–4 months	Minimal clinical activity against hormone refractory prostate cancer was observed	No (PSA and disease progression)
Paur et al.^95^	79 patients with non‐metastatic prostate cancer	Localised 100%	Dietary supplementation—intervention with either (1) tomato products containing 30‐mg lycopene per day; (2) tomato products plus selenium, omega‐3 fatty acids, soy isoflavones, grape/pomegranate juice and green/black tea (tomato‐plus); or (3) control diet Duration: 3 weeks	Intervention with tomato products alone or in combination with selenium and n‐3 fatty acids lowered PSA. Effect may depend on both aggressiveness of the disease and the blood levels of lycopene, selenium and omega‐3 fatty acids.	Yes (PSA)
Kumar et al.^96^	42 men with localised PCa	Localised 100%	Dietary supplementation—supplemented with 15, 30 or 45 mg of lycopene or placebo from biopsy to prostatectomy Duration: 1 month	No clear effects on PSA.	No (PSA)
Guess et al.^97^	10 men with PCa	Localised 100%	Dietary supplementation—effect of modified citrus pectin (Pecta‐Sol) after localised treatment Duration: 12 months	The PSA doubling time increased (*p* value < 0.05) in 7 (70%) of 10 men after taking modified citrus pectin (MCP) for 12 months compared with before taking MCP	Yes (PSA doubling time)
Pantuck et al.^98^	46 patients	Localised 100% (including locally advanced but all N0 and M0)	Dietary supplementation—patients were treated with 8 oz of pomegranate juice daily after localised treatment (wonderful variety, 570‐mg total polyphenol gallic acid equivalents) until disease progression Duration: Up to 24 months	Mean PSA doubling time significantly increased from a mean of 15 months at baseline to 54 months post treatment (*p* < 0.001).	Yes (PSA doubling time)
Paller et al.^99^	104 patients with rising PSA post primary treatment	Localised 100%	Dietary supplementation—1 g or 3 g of pomegranate extract after localised treatment Duration: up to 18 months (92% 6 months, 70% 12 months, 36% 18 months)	Intervention was associated with ≥6‐month increases in PSA doubling time in both treatment arms without adverse effects.	Yes (PSA doubling time)
Pantuck et al.^100^	183 patients with rising PSA levels after primary therapy for PCa.	Localised 100%	Dietary supplementation—pomegranate extract or placebo Duration: up to 36 months	Compared with placebo, pomegranate extract did not result in a significant prolongation of PSA doubling times in PCa patients with rising PSA after primary therapy	No (PSA doubling time)
Stratton et al.^101^	140 men with PCa on active surveillance	Localised 100%	Dietary supplementation—two different doses of high selenium yeast and placebo Duration: Up to 5 years	Intervention did not show a protective effect on PSA velocity in subjects with localised prostate cancer.	No (PSA velocity)
Lazarevic et al.^102^	54 patients with localised PCa prior to radical prostatectomy	Localised 100%	Dietary supplementation—genistein or placebo (e.g., soy beans or fava beans). Duration: 3–6 weeks	Intervention reduced the level of serum PSA in patients with localised PCa, without any effects on hormones.	Yes (PSA)
Hamilton‐Reeves et al.^103^	86 patients with localised PCa prior to prostatectomy	Localised 100%	Dietary supplementation—soy isoflavone capsules (80 mg/day of total isoflavones, 51 mg/day aglucon units) Duration: 6 weeks	Changes in serum total oestrogen, total testosterone, free testosterone, PSA, estradiol and total cholesterol were not significantly different between intervention and placebo arms	No (PSA)
deVere White et al.^104^	53 men with PCa on active surveillance	Localised 100%	Dietary supplementation—450‐mg genistein, 300‐mg daidzein and other isoflavones daily. Duration: 6 months	Intervention did not lower PSA levels in men with low‐volume PCa.	No (PSA)
Pendleton et al.^105^	20 PCa patients with rising PSA after prior local therapy	Localised 100%	Dietary supplementation—patients were given soy milk containing 47 mg of isoflavonoid per 8 oz serving three times per day. Duration: 12 months	Slope of PSA after study was significantly lower than that before study in 6 patients, was significantly higher than before study entry in 2 patients and for the remaining 12 patients, the change in slope was insignificant.	No (PSA)
Bosland et al.^106^	177 men at high risk of PCa recurrence after radical prostatectomy	Localised 100%	Dietary supplementation—supplement of soy protein isolate was started within 4 months after surgery and continued for up to 2 years Duration: Up to 2 years	Supplementation with soy protein isolate for 2 years post radical prostatectomy did not reduce biochemical recurrence	No (PSA)

Abbreviations: BCR, biochemical recurrence; HGF, hepatocyte growth factor; IMRT, intensity modulated radiation therapy; MHSs, men's health supplements; PCa, prostate cancer; PSA, prostate specific antigen.

**TABLE 3:** Summary of 16 research studies looking at the influence of various dietary, physical activity and lifestyle interventions on putative inflammatory and immune biomarkers for PCa comprising 750 total participants.

Reference	Cohort size and demographics	Type of lifestyle intervention	Biomarker activity outcomes
Schauer et al.^107^	20 patients with early stage PCa awaiting prostatectomy	Physical activity	Exercise mobilises NK, CD8 T and NKT‐like cells in patients with prostate cancer
			After exercise, NK cytotoxic activity increases against K562 and LNCaP but not PC3
			Markers of inflammation inversely correlate with exercise‐enhanced immune function.
Penedo et al.^108^	192 men with advanced PCa (stages III–IV)	Lifestyle counselling or stress management	Inflammatory markers decreased among men in stress management and lifestyle counselling
			Decreases in inflammatory markers were not sustained at 12 months
Galvão et al.^109^	57 men with PCa undergoing ADT	Physical activity	Significantly decreased levels of CRP
Hoyt et al.^110^	41 men with localised PCa that had undergone prostatectomy or radiation therapy	Emotional expression and processing	Emotional processing predicted lower inflammatory markers IL‐6, sTNF‐RII and CRP.
			Emotional expression was significantly associated with higher levels of sTNF‐RII.
			Expression of emotion may be associated with higher inflammation only in the context of low emotional processing.
Djurhuus et al.^111^	30 men with localised PCa	Physical activity	One exercise session on the day prior to radical prostatectomy is insufficient to effect NK cell infiltration into prostatic tissue.
Schenk et al.^112^	22 male patients with intermediate or high‐risk PCa scheduled for radical prostatectomy	Physical activity	Physical exercise mobilises and redistributes NK cells, particularly those with cytotoxic phenotype.
			No increase of NK cell tumour infiltration.
Djurhuus et al.^113^	30 patients with localised PCa undergoing radical prostatectomy	Physical activity	The per‐protocol analysis (HIIT 4 times per week) showed a significant increase in tumour NK‐cell infiltration.
			The total number of training sessions was positively correlated with the change in NK‐cell infiltration
Kaushik et al.^114^	29 men with newly diagnosed PCa	Physical activity/stress management (yoga)	Increased numbers of circulating CD4+ and CD8+ T‐cells and production of interferon‐gamma by natural killer cells.
			Increased Fc receptor III expression in natural killer cells.
			Decrease in numbers of regulatory T‐cells, myeloid‐derived suppressor cells, suggesting antitumour activity
			Reduction in inflammatory cytokine levels (granulocyte colony‐stimulating factor, monocyte chemoattractant protein and FMS‐like tyrosine kinase‐3 ligand
Hanson et al.^115^	11 men with PCa on ADT, 14 men with PCa not on ADT and 8 healthy controls	Physical activity	PCa survivors have NK cell mobilisation comparable with healthy controls following acute exercise.
			CD56 total cell egress tended to be attenuated in ADT.
			ADT also consistently showed a less mature phenotype with greater proportion of IFNγ expression and possibly lower cytotoxicity. Though perforin levels remained unchanged.
			The proportion of perforin expressing NK cells in PCa was reduced, suggesting these cells may be more prone to degranulation. By 24 h, all NK and leukocyte populations returned to baseline levels, suggesting that consecutive training sessions could be used without adverse immune system effects.
Ornish et al.^116^, ^117^	30 men with low risk PCa	Lifestyle	A significant increase in telomerase activity and consequently telomere maintenance capacity in human immune‐system cells was observed.
Hojan et al.^61^, ^118^	72 intermediate and high‐risk PCa patients undergoing radiation therapy	Physical activity	A decrease in the levels of proinflammatory cytokines was observed.
Hvid et al.^119^	25 patients with biochemical recurrence of PCa or on active surveillance	Physical activity	The intervention group showed significant improvements in plasma triglycerides, IGF‐1, IGFBP‐1, adiponectin and fasting glucose levels.
			No changes were observed for insulin sensitivity, cholesterols, testosterone, fasting insulin, plasma TNF‐alpha, IL‐6 or leptin levels.
Galvão et al.^120^	10 PCa patients on ADT for at least 2 months	Physical activity	Serum growth hormone (GH), dehydroepiandrosterone (DHEA), interleukin‐6, tumour necrosis factor‐alpha and differential blood leukocyte counts increased following acute exercise.
Cohen et al.^121^	159 PCa patients scheduled for radical prostatectomy	Stress management/supportive attention	Men in the stress management group had significantly higher levels of natural killer cell cytotoxicity and circulating proinflammatory cytokines 48 h post‐surgery than men in the supportive attention group and higher levels of natural killer cell cytotoxicity and IL‐1β than men receiving standard care.

Abbreviations: ADT, androgen deprivation therapy; HIIT, high intensity interval training; PCa, prostate cancer.

## 5 SPECIFIC EFFECT OF LIFESTYLE INTERVENTIONS ON MENTAL HEALTH IN PCa PATIENTS

**FIGURE 3:**
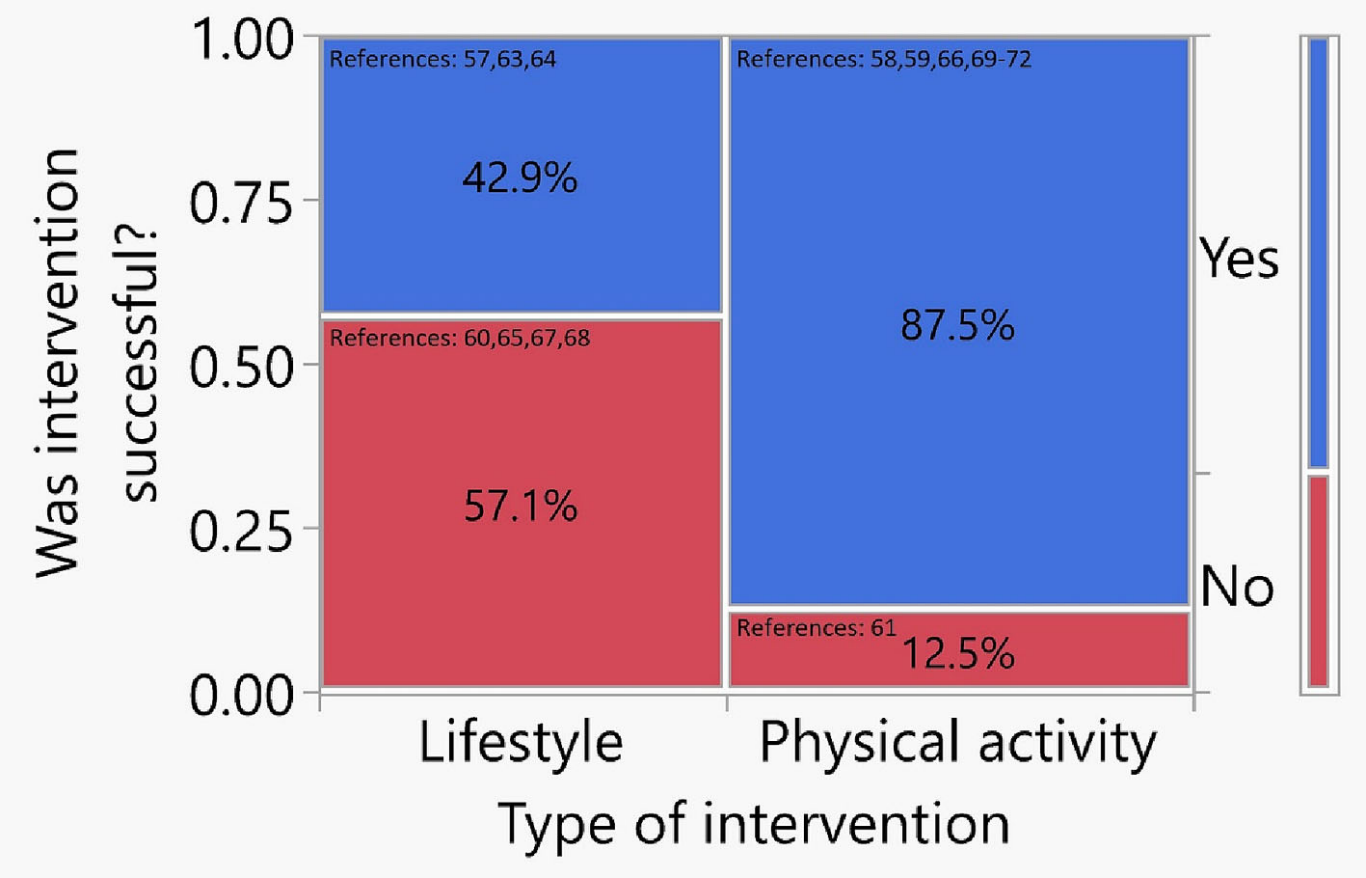
Mosaic plot representing the proportions of successful interventions by type on mental health outcomes for prostate cancer (PCa) patients for all studies summarised in Table 1.

For the increasing survivorship of PCa patients, balancing a positive mental outlook in the present against worries for the future is crucial,^122^ and there is an unmet need for mental health services within PCa patients' broader oncological care.^123^ Not surprisingly, PCa patients are more likely to be prescribed antidepressants compared with their cancer‐free counterparts, and some studies have reported suicide rates up to 6.5 times higher in PCa patients compared with controls.^124^ There have been several studies investigating the effect of lifestyle interventions on mental health in PCa patients or cohorts including a significant fraction of PCa patients. A summary of these is provided in Table 1 with the results represented in Figure 3.

As is evident from Table 1, cohorts and outcome assessments are heterogenous making uniform conclusions challenging. There are 15 studies presented in Table 1 incorporating approximately 3000 patients with PCa. In Figure 3, the schematic mosaic plot reflecting these studies suggests those with a focus on PA may be more likely to have a positive influence on mental health.

### 5.1 Studies showing a positive influence on mental health

Of the 10 studies demonstrating a positive influence on mental health, 2 focused on broader interventions combining PA with diet and stress management and 1 on a more esoteric approach using a smart shirt and mobile app solution for depression self‐management.^46^, ^52^, ^53^ One of the multi‐factor lifestyle interventions combined a low‐fat vegan diet with 3 h per week of moderate PA and 1 h per week of MSBR.^52^ Recruits all had localised PCa and adhered to the programme for 12 months, with their mental health assessed at the end of the intervention by SF‐36 form, the perceived stress scale and the sexual function subscale of the UCLA PCa index. Based on this assessment, recruits reported better mental and physical health related quality of life (HR‐QOL), sexual function and decreased perceived stress.^52^ Baguley et al.^53^ also combined diet and PA, but their participants had advanced PCa on androgen deprivation therapy (ADT). Adhering to a Mediterranean diet with high intensity interval training (HIIT) for 20 weeks, recruits had better cardiorespiratory fitness, reduced body weight and better QOL and cancer related fatigue as assessed by the Functional Assessment of Chronic Illness Therapy (Fatigue (FACIT‐F), FACIT‐general (FACIT‐G) questionnaires) and SF‐36 form.^53^ The Smart Shirt and Mobile App intervention was performed on patients with different cancers, and 24% of whom had stages II, III or IV PCa. They used the technology for 12 weeks reporting significantly lower depressive symptoms at the end of the intervention (*p* < 0.001), as assessed by Beck Depression Inventory II Questionnaire.^46^

Of the 10 studies demonstrating a benefit to mental health, 7 focused on PA alone. Conroy et al. examined breast and PCa patients (whose disease stages were not available) as a part of a secondary analysis of RENEW Trial patients focused on obesity, finding those who successfully completed moderate to vigorous PA (>3 metabolic equivalent [MET] hours per week) demonstrated improved emotional role functioning, mental health and vitality as assessed by the SF‐36.^47^ Farris et al.^48^ investigated 817 PCa patients (74.1% and 15.9% with localised and advanced disease respectively) assessing their PA at 3 separate time points over 36 months from diagnosis and found those who adhered to the guidelines of moderate to vigorous PA (150 min moderate or 75 min vigorous per week) reported better QOL scores as assessed by the SF‐36. The other 5 successful PA‐focused studies examined predominantly patients with localised PCa with PA interventions of 2–6 months including regimes of 3 weekly sessions of resistance exercise training using resistance bands, combined group and individual sessions 4 times per week, supervised HIIT programmes, training twice a week at a local football club and moderate PA 5 days per week.^47^, ^48^, ^55^, ^58^, ^59^ Mental health assessment metrics were evaluated using several different validated measurement tools (see Table 1.).^47^, ^48^, ^55^, ^58^, ^59^

### 5.2 Studies showing no influence on mental health

Of the 5 studies in Table 1 failing to demonstrate a positive influence on mental health, 4 examined combined Lifestyle programmes and 1 PA alone. One of the combined lifestyle studies was based on an analysis of baseline assessments for PCa patients over 65 years more than 5 years from diagnosis from the RENEW trial.^49^ They found patients who engaged in more weekly minutes of exercise had better physical QOL, physical functioning and vitality but, apart from better social functioning, no improvement in mental health assessed by the SF‐36. Three other studies examining Lifestyle counselling and psychosocial support (with pelvic exercises) without any specific dietary or PA factors failed to demonstrate any improvements in mental health in either localised or advanced PCa patients.^54^, ^56^, ^57^ The duration of these was 5–6 months, and the mental health assessment tools included SF‐12, the Patient Health Questionnaire (PHQ‐9; depression screening), the Attention Functional Index (AFI; cognitive function) and the Lee Fatigue Scale.^54^, ^56^, ^57^ The one negative PA alone intervention study was on advanced PCa patients undergoing daily moderate activity.^50^ After 6 months, the PA group failed to demonstrate improved mental health when compared with an educational support group and a standard care group, although a sub‐group analysis did show improvement in those with limited psychosocial functioning at baseline. The assessment tools included the SF‐36 as well as the Centers for Epidemiologic Studies‐Depression (CES‐D) and State/Trait Anxiety Inventory scale (STAI).^50^, ^51^

The molecular mechanism behind which exercise improves mental health may relate to increasing serotonin, endothelial growth, insulin‐like growth and brain‐derived neurotrophic factors within the central nervous system (CNS).^125^ Reducing obesity, stress and systemic inflammation may have similar neurophysiological effects on the CNS that are advantageous for mental well‐being, but further research is required.

Making clear PCa‐specific guidelines for lifestyle interventions to improve mental health is challenging based on the current evidence. Well‐designed randomised controlled trial (RCTs) are imperative, some of which are already under way with results pending.^126^ Although both SF‐36 and QOL questionnaires used in the studies reviewed have validated mental health and well‐being domains, some studies may prefer FACT‐P for assessment of PCa patients, and uniformity in outcome assessment would make trial comparisons more meaningful. Developing collaborations with mental healthcare professionals will also be important, not only to provide insight into the treatment of mental health disorders but also to facilitate trial recruitment.^127^

## 6 SPECIFIC EFFECT OF LIFESTYLE INTERVENTIONS ON DISEASE OUTCOMES IN PCa PATIENTS

**FIGURE 4:**
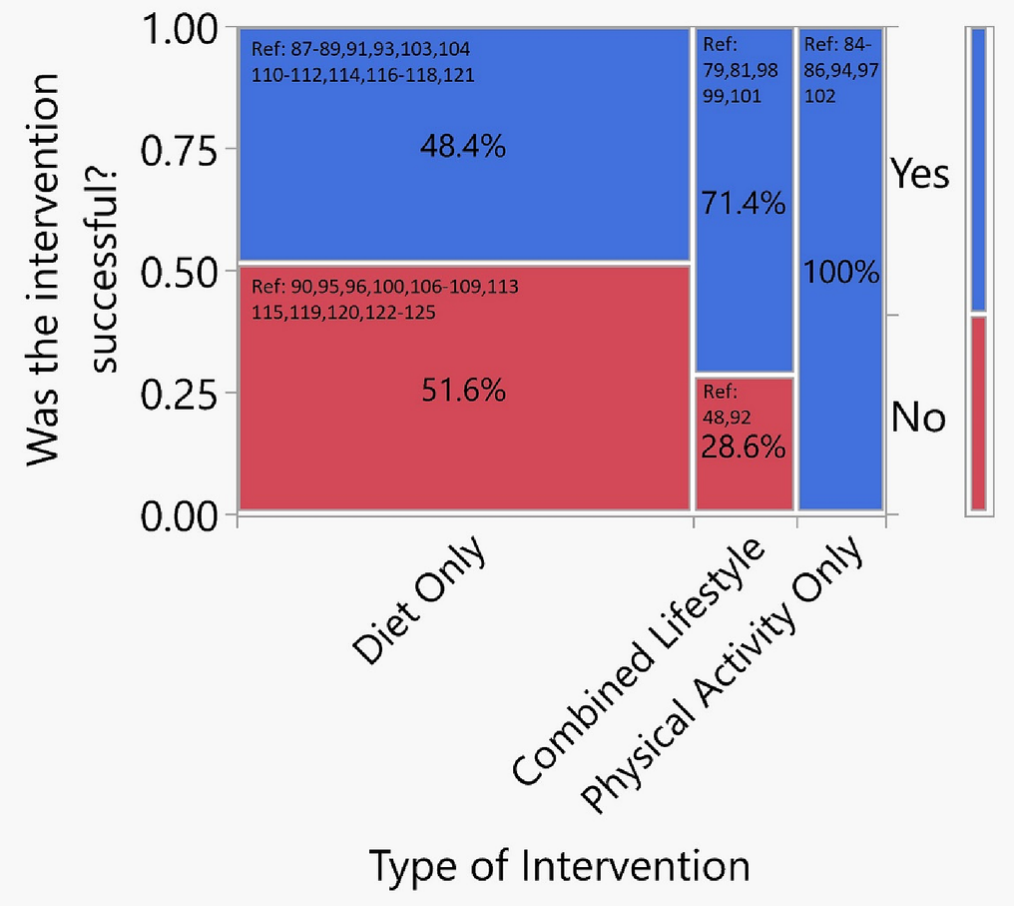
Mosaic plot representing the proportions of successful interventions by type on prostate cancer oncological outcomes for prostate cancer (PCa) patients for all studies summarised in Table 2. The width of each column is proportional to the number of studies for each intervention type. Bar on right represents the entire group or the success rate of all lifestyle and physical activity interventions.

Numerous studies have examined the effect of lifestyle interventions on oncological outcomes in PCa patients, and partly as a result, healthy lifestyle guidelines have been issued by the National Comprehensive Cancer Network (NCCN).^128^ An early landmark study investigated a multifactor lifestyle programme that required a vegan diet (with 10% of calories from fat) supplemented by vitamin C (2 g daily), soy, vitamin E (400iu daily), fish oil (3 g daily) and selenium (200 mcg daily).^129^ Other factors in the programme included yoga, progressive relaxation, breathing and mediation, with PA from walking 30 mins × 6 days a week, and a weekly support group to provide advice and support on how to adhere to what became known as the ‘Ornish protocol’.^129^, ^130^ This programme was found to slow disease progression in a group of 93 patients using PSA values and the growth of LNCaP cancer cells in culture as study end points. PSA decreased by 4% in the intervention group, whereas a 6% increase was observed in the control group (*p* = 0.016). The growth of LNCaP PCa cells was inhibited by a factor of 8 using serum from the experimental group compared to the controls (*p* < 0.001), a result reproduced in several studies.^129^, ^131^, ^132^ A diet and MBSR intervention was also found to lower the rate of PSA increases as well as their absolute values in cases of biochemically recurrent PCa.^39^, ^63^ Lifestyle interventions have been shown to reduce upgrading in AS biopsies and treatment conversion rates,^62^, ^133^ as well as potentially improving outcomes in those who do convert to treatment.^134^ Table 2 summarises 44 research studies looking at the influence of various dietary, PA and lifestyle interventions on PCa oncological outcomes comprising over 36 000 total participants. Once again, these studies are heterogenous in the lifestyle changes recommended as well as their duration and patient populations. A mosaic plot representing these studies is presented in Figure 4.

### 6.1 Diet only

There are 31 studies focused on diet, 48.4% (15) of which were successful at improving oncological outcomes^67^, ^68^, ^69^, ^71^, ^73^, ^84^, ^85^, ^91^, ^92^, ^93^, ^95^, ^97^, ^98^, ^99^, ^102^ and 51.6% (16) of which were not.^70^, ^75^, ^76^, ^80^, ^87^, ^88^, ^89^, ^90^, ^94^, ^96^, ^100^, ^101^, ^103^, ^104^, ^105^, ^106^ Most of the diet only studies showing improved oncological outcomes were on PCa patients with localised (non‐metastatic) disease except for two studies, one of which 34% of the cohort had advanced disease^69^ and the other of which all 20 patients had metastatic hormone refractory PCa.^91^

In total for the 15 successful diet only studies, 5198 patients had localised (non‐metastatic) disease, and only 34 patients had advanced disease. The dietary interventions included lycopene supplementation (for 3 weeks prior to surgery,^67^, ^85^ for 3 months alone^91^ and with or without selenium or omega 3 fatty acids for 3 weeks prior to surgery^95^); high phytoestrogens (soy and linseed for 24 days before surgery^68^); soy and isoflavones supplementation (for 5.5 months in patients with rising PSA levels post treatment for localised disease [66%] or with advanced disease [34%]^69^ or 3–6 weeks prior to surgery^102^); combined supplementation of soy, isoflavones, lycopene, silymarin and antioxidants (for 10 weeks in patients with biochemical recurrence [BCR] post treatment^71^); replacing animal fat with vegetable fat^73^; green tea (for 1 month prior to surgery^92^); polyphenol E (for 1 month prior to surgery^93^); citrus pectin (for 12 months after primary treatment for localised disease^97^); and pomegranate (up to 24 months after primary treatment for localised disease^98^, ^99^). Oncological outcomes measured were PSA levels, PSA velocity, PSA doubling time, Free/Total PSA ratios, tumour size, surgical margins and cancer cell proliferation rates on surgical pathology, hepatocyte growth factor (HGF) and VEGF levels as well as PCa‐specific and all‐cause mortality.^67^, ^68^, ^69^, ^71^, ^73^, ^84^, ^85^, ^91^, ^92^, ^93^, ^95^, ^97^, ^98^, ^99^, ^102^

The diet only studies that were unsuccessful again consisted of mainly patients with localised disease^70^, ^75^, ^76^, ^80^, ^87^, ^88^, ^96^, ^100^, ^101^, ^103^, ^104^, ^105^, ^106^ with only three studies on patients with advanced disease.^89^, ^90^, ^94^ The 16 unsuccessful studies consisted in total of 3968 patients with localised disease and only 67 patients with advanced disease. The dietary interventions were isoflavones supplements from red clover (given for 20 days pre surgery^70^), marketed Men's and Prostate Health Supplements (e.g., vitamins, minerals and herbs^75^), reduced alcohol consumption,^76^ sodium salicylate supplementation with or without vitamin C and copper and manganese gluconates (given for 12 months^80^), lycopene supplementation (for 12 months post treatment in those with BCR,^87^ for 3 weeks before diagnostic biopsy,^88^ for 4–6 months in advanced disease patients^89^, ^90^ or for month prior to surgery^96^), green tea (for 2–4 months in patients with advanced disease^94^), pomegranate (for up to 36 months post localised disease treatment^100^), selenium (for up to 5 years for AS patients^101^) and soy isoflavones in different forms (for 6 weeks prior to surgery,^103^ for 6 months in AS patients^104^ or for 12–24 months after treatment for localised disease^105^, ^106^). The outcomes measured in the diet only studies showing no influence were apoptosis and surgical pathology downgrading, PSA levels, PSA velocity and doubling time, metastatic progression, PCa‐specific survival and diagnostic prostate biopsy DNA oxidation and lipid peroxidation products.^70^, ^75^, ^76^, ^80^, ^87^, ^88^, ^89^, ^90^, ^94^, ^96^, ^100^, ^101^, ^103^, ^104^, ^105^, ^106^

### 6.2 Combined lifestyle intervention

There are seven studies focused on combination lifestyle interventions, five of which showed a positive impact on oncological outcomes and two of which did not. The five successful studies included the 2‐year follow up for the Ornish programme for localised PCa patients in AS, whose intervention was low fat vegan diet, PA (3 h per week of moderate exercise) and MBSR (1 h per week) for a duration of 12 months (discussed above).^62^ At 2‐year follow up, they showed a significant reduction in PSA and disease progression with concomitant reduction in conversion to active treatment.^62^ The other four successful studies included a plant based diet with stress management, and retrospective sub analyses of the Health Professions Follow Up, CAPSURE and PROTECT studies examining the influence of BMI reduction with healthy diet, vigorous PA and non‐smoking, as well as inflammatory, hyperinsulinemia and insulin resistant diets. Follow up ranged from 22 months to 27 years, and outcome measures were PSA levels, PSAD times, metastatic progression and PCa‐specific mortality.^63^, ^78^, ^79^, ^81^ For all these five successful studies, the total number of patients was 8015 with both localised and advanced disease although the exact proportions are not available.^62^, ^63^, ^78^, ^79^, ^81^ The two studies examining combined lifestyle interventions that failed to show any influence on PCa oncological outcomes included one intervention of a diet of 170 g/day of whole grain rye with 45‐min vigorous activity per week for 6 months in 26 AS localised PCa patients and another 12‐week programme of aerobic and resistance exercise with dietary advice in 50 advanced PCa patients on ADT.^72^, ^82^

### 6.3 PA only

Six studies focused on PA only interventions, and all demonstrated a positive influence of PCa outcomes. One of these studies was prospective examining 12 weeks of supervised HIIT on a treadmill 3 times a week in 52 localised PCa AS patients.^83^ Post intervention, patients in the programme group not only had lower PSA levels and velocity but also incubation with their serum resulted in decreased LNCaP cellular growth.^83^ In the other five studies, including sub analyses of CAPSURE and Health Professions Follow Up Studies, patients received questionnaires focused on their PA habits and were followed for up to 27‐year post recruitment.^64^, ^65^, ^66^, ^74^, ^77^ They demonstrated improvements in oncological outcomes including BCR, metastatic progression, PCa‐specific and overall mortality in patients with predominantly localised and advanced PCa (total number of patients = 9698).

## 7 SPECIFIC EFFECT OF LIFESTYLE INTERVENTIONS ON BIOMARKERS, METABOLIC IMAGING, SURGICAL PATHOLOGY AND GENETICS IN PCa PATIENTS

Given that systemic inflammation has been implicated in PCa oncogenesis and poorer oncological outcomes, it seems reasonable to explore inflammatory biomarkers as indicators of its severity and improvement. Increased monocyte to lymphocyte ratios (MLRs), including neutrophil to lymphocyte ratio (NLR) and platelet to lymphocyte ratio (PLR), have been shown in some studies to predict BCR, disease progression and overall survival in PCa,^20^, ^135^, ^136^, ^137^, ^138^, ^139^, ^140^ as well as being under the influence of sociodemographics, lifestyle, diet and PA.^141^, ^142^, ^143^ Easily measured from a complete blood count (CBC), elevated NLRs, PLRs and another similarly derived marker SII (the systemic immune‐inflammation index − neutrophil count × platelet count/lymphocyte count) have all been found significantly correlated to obesity and BMI.^144^ However, results examining their clinical utility have been conflicting. A retrospective study from Japan on 633 men before PCa diagnosis found, by contrast, low NLR and PLR were predictive of finding PCa at subsequent biopsy.^145^ In other studies, the accuracy of NLR as a prognostic marker of disease progression in localised PCa or response to treatment (e.g., docetaxel, cabazitaxel or radium‐223) in advanced PCa has been inconclusive suggesting the need for further study.^146^, ^147^, ^148^, ^149^

Investigating specifically SII in a retrospective multicentre cohort of over 6000 men treated surgically for localised PCa, elevated presurgical SII was found to be significantly associated with both adverse surgical pathology and longer term BCR.^150^ The predictive value of the SII is supported by other studies in localised PCa post surgery examining not only BCR but also long term survival.^151^, ^152^, ^153^

Focusing on PCa oncogenesis, Fowke and Motley^154^ studied links between metabolic dysregulation, obesity and systemic inflammation in 160 men with high grade prostatic intraepithelial neoplasia (HGPIN), regarded in high volume as a PCa risk factor, who were then diagnosed with PCa at subsequent biopsy. They found statin use was linked to overall PCa diagnosis, but obesity parameters and markers of systemic inflammation were not. In another study, no clear relationship was found between systemic inflammatory or oxidative stress markers in blood or urine with prostate size or lower urinary tract symptoms (LUTS) in patients with BPH but not PCa.^155^ By contrast, an earlier study examining data from the PCa Prevention Trial, an RCT assessing whether Finasteride reduced PCa risk, found markers of systemic inflammation (in this case c‐reactive protein) or lower levels of soluble receptors that bind inflammatory cytokines (such as TNF and IL‐6) did increase BPH risk.^156^ The inference of these studies of HGPIN and BPH is that they may represent overall ‘prostate health’ and draw on the belief that the path to PCa is via histological inflammation with BPH included in this process. These results are, however, inconclusive.

Studies have also assessed inflammatory cytokines as biomarkers of PCa oncological outcomes. In a retrospective analysis of 237 men undergoing surgery for localised PCa, high TNF‐α levels were found to be positively correlated with upgraded Gleason scores post surgery, whereas, counterintuitively, high levels of serum IL‐6 were found to be positively related with Gleason score downgrades.^157^

In another study, men were randomly selected from a prospective cohort derived from the Osteoporotic Fractures in Men (MrOS) study, with a view to investigating correlation between serum inflammatory cytokine levels and risk of subsequent PCa. After 6 years of follow up, IL‐10 levels were found to be associated with lower risk of PCa, but there was no association with PCa risk and either TNF alpha or IL‐6.^158^

Studies where biomarkers of inflammation or immunity were tracked as a function of intervention are summarised in Table 3. Some degree of success was observed in the majority, but there are fewer participants compared with the studies on oncological and mental health outcomes. These studies are presented by way of summary and for reference to highlight emerging putative biomarkers that may have clinical utility in the future.

## 8 CONCLUSION

Making specific recommendations on lifestyle interventions for PCa patients is difficult based on the current evidence, which stems sometimes from studies not only from different cancers but also those using diverse intervention protocols and assessment tools. Nevertheless, notwithstanding this heterogeneity of patient populations and interventions, the evidence that a reduction in body fat with improved diet and PA may reduce the risk of PCa and improve both mental health and oncological outcomes is compelling, especially when interventions include moderate to vigorous PA. Clinical evidence for dietary supplements is also inconsistent, although some may have promise alone or in combination. With this is in mind, leading organisations including the National Cancer Institute, the National Institute of Clinical Excellence (UK), the American Cancer Society and the Center for Disease Control are all advocates of diet and exercise for PCa prevention and management as a part of broader oncological care. However, more research is needed. Using the analogy of diabetes as a metabolic disease, the use of diet to manage prediabetes and the clinical utility of HbA_1C_ as a biomarker, future randomised clinical trials will hopefully inform clinicians with more detail on the specifics of diet, PA and MBSR interventions before and after PCa diagnosis to improve both well‐being and prognosis. The use of CBC‐derived inflammatory biomarkers may have some promise, and although it is attractive to suggest inflammatory cytokines have utility given the proposed links of obesity to systemic inflammation, a deeper understanding of their molecular biology in relation to obesity and PCa oncogenesis is required before this becomes a reality.

## AUTHOR CONTRIBUTIONS

Dr Zach Dovey: literature search and PRISMA methodology, writing the manuscript, tables, figures and editing. Dr Nikhil Waingankar: PRISMA methodology, editing the manuscript. Dr Amir Horowitz: editing the manuscript.

## CONFLICT OF INTEREST STATEMENT

Dr. Zach Dovey is Medical Director and stock owner (with certificate of shares) of Medtech Holdings Ltd.

## Supporting information


**Figure S1:** Plot of p‐values representing the significance (or not) on the outcomes of components of the SF‐36 survey as a result of lifestyle interventions.

**Figure S2:** Forest plots assessing the influence of the extent of vigorous PA on all cause mortality in prostate cancer patients.
