# Supplementary material for: The influence of lifestyle changes (diet, exercise and stress reduction) on prostate cancer tumour biology and patient outcomes: A systematic review
Source: BJUI Compass. 2023 Apr 6;4(4):385–416. doi: 10.1002/bco2.237 (PMC10268595; doi:10.1002/bco2.237)
Supplement: Supplementary file 1 — Figure S1: Plot of p‐values representing the significance (or not) on the outcomes of components of the SF‐36 survey as a result of lifestyle interventions. [file BCO2-4-385-s002.docx]

Supplemental Figure 1: Plot of p-values representing the significance (or not) on the outcomes of components of the SF-36 survey as a result of lifestyle interventions.


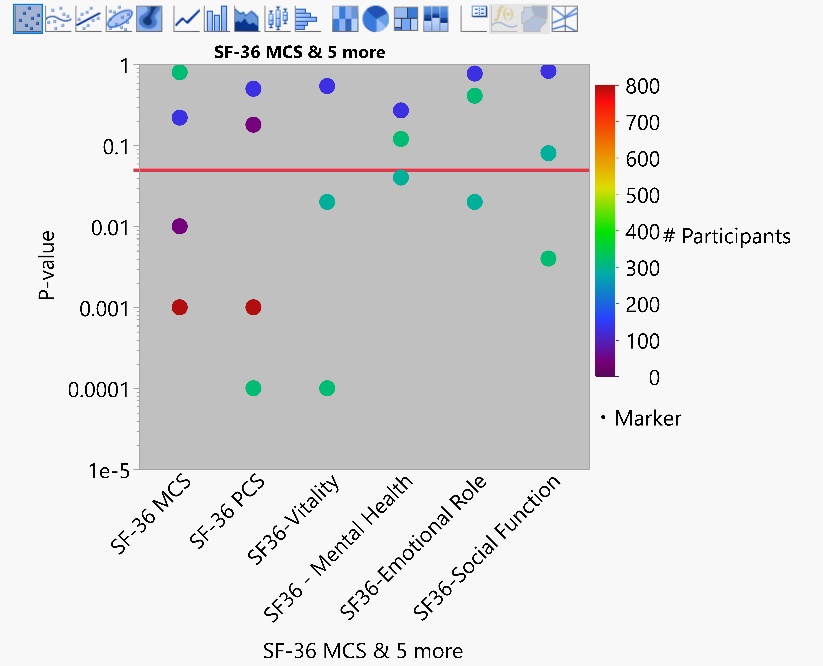


Examining those studies that specifically used the SF-36 form alone to highlight any meaningful trends is also complex. Supplemental Figure 1. is a plot of distributions of p-values reflecting the significance of an intervention on the SF-36 scores. The number of studies used to assemble the curve is low, and the number of p-values below (9) the horizontal red line value of 0.05 (horizontal red line) is less than those above (11). However, those falling below the red line are from studies with higher participation which thus have more statistical power. For this subanalysis, there was no clear relationship between studies showing significance as to whether the intervention was diet only, physical activity only, or both. Also, the stage of cancer was not reported in all studies, although in the study that reported a highly significant positive influence,^59^ 77% of the cohort had localised disease (62% of whom had Gleason scores greater than 7 and so would be offered treatment). The report of Daubenmier et al^63^ that also showed significant improvement in the SF36-MCS scores as a result of their diet and PA intervention used patients with low risk disease in active surveillance.

The collective group of successful trials in Table 1 would suggest interventions including PA are more likely to result in improvements in mental health. Unfortunately, this sub analysis of studies using the SF-36 form alone, which attempts to eradicate any bias arising from different mental health assessment tools, does not provide any meaningful trends as to which interventions are more effective in which patients.
