# Supplementary material for: The influence of lifestyle changes (diet, exercise and stress reduction) on prostate cancer tumour biology and patient outcomes: A systematic review
Source: BJUI Compass. 2023 Apr 6;4(4):385–416. doi: 10.1002/bco2.237 (PMC10268595; doi:10.1002/bco2.237)
Supplement: Supplementary file 2 — Figure S2: Forest plots assessing the influence of the extent of vigorous PA on all cause mortality in prostate cancer patients. [file BCO2-4-385-s001.docx]

Supplemental Figure 2: Forest plots assessing the influence of the extent of vigorous PA on all cause mortality in prostate cancer patients.


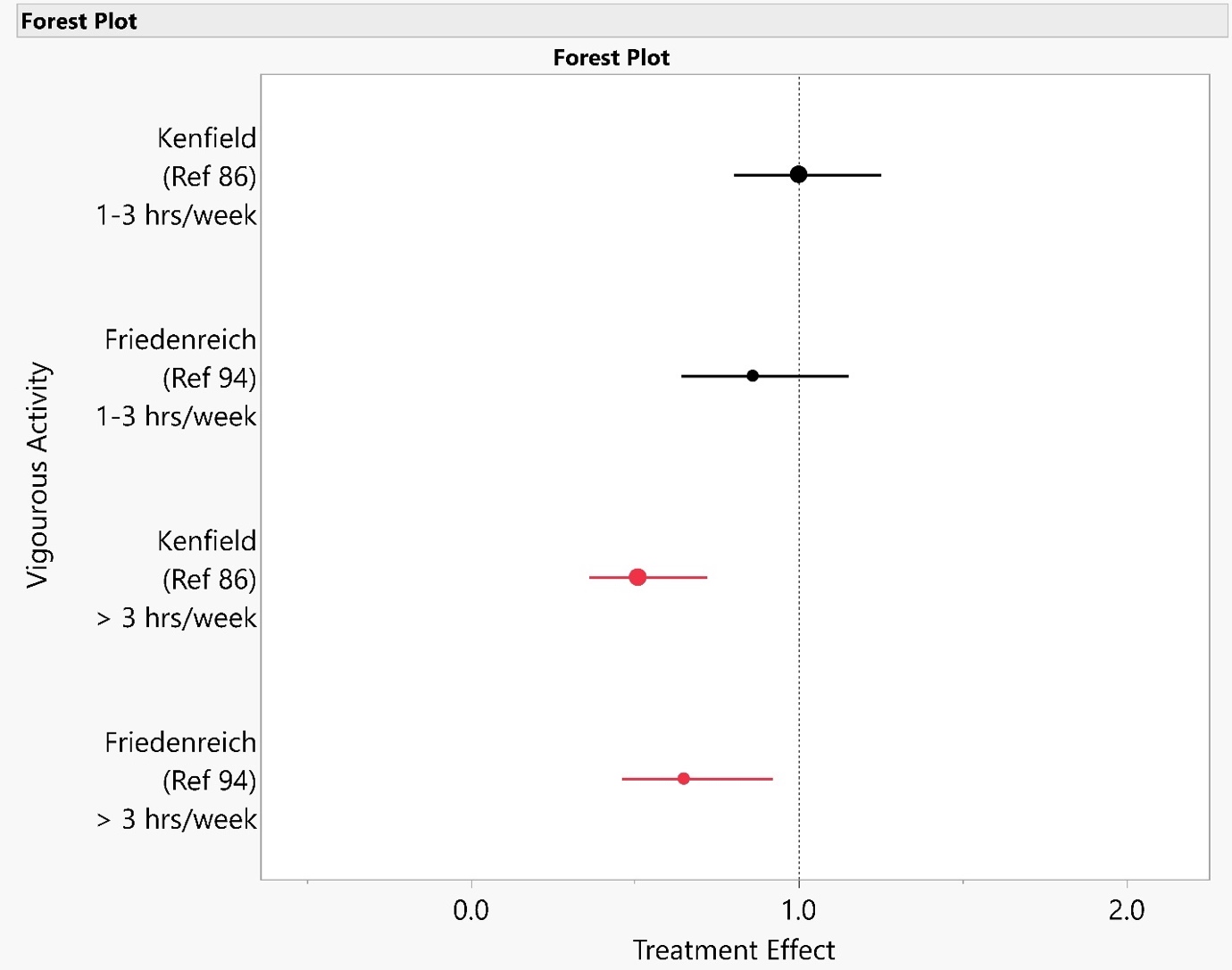


A sub-analysis of the studies focused on PA alone can be seen in the Forest plot shown in Supplemental Figure 2 assessing the influence of vigorous PA on the hazard ratios for all-cause mortality. These studies were chosen based on the statistical analysis they used totalling more than 3500 PCa patients who had spent 1-3 hours a week engaging in vigorous PA as a part of the intervention. Looking at the Forest plots of these 2 cohorts, both fail to show significance, but the red points represent those in the cohort that engaged in more than 3 hours of vigorous activity per week, which then does show significance. This result may be of interest for future trail design but clearly more research is needed to clarify what kind of PA and how much is required per week for the optimum effect.
